# Deep Learning for Protein Secondary-Structure Segmentation in Intermediate-Resolution Cryo-EM Maps: A Resolution-Stratified Benchmark Dataset and Controlled Evaluation

**DOI:** 10.1021/acsomega.6c06887

**Published:** 2026-10-15

**Authors:** Md Ehashan Rabbi Pial, Farhan Noor Dehan, Salim Sazzed

**Affiliations:** Department of Computer Science, 7604 Georgia Southern University, Statesboro, Georgia 30458, United States

## Abstract

Accurate segmentation of protein secondary structures in cryo-electron microscopy (cryo-EM) maps is crucial for protein tracing and atomic model building, yet remains challenging at intermediate resolutions. Although deep learning architectures have recently been applied to this task, there remains a lack of standardized evaluation protocols and systematic resolution-stratified analyses. Consequently, it remains unclear how segmentation performance varies across finer resolution ranges within the intermediate-resolution regime and how different volumetric segmentation architectures compare under controlled experimental conditions. To address these gaps, we present a nonredundant, resolution-stratified benchmark dataset and a controlled evaluation framework for protein secondary-structure segmentation in intermediate-resolution cryo-EM maps. The dataset comprises 972 nonredundant protein-chain samples from 495 cryo-EM protein structures resolved at 4.5–8.0 Å, with voxel-level annotations for helices, sheets, and non-periodic secondary-structure (NPSS) regions. The dataset is stratified into three resolution ranges—4.5–5.5, 5.5–6.5, and 6.5–8.0 Å—to enable systematic analysis of resolution-dependent segmentation performance. Using this benchmark, we evaluate five representative convolutional and transformer-based volumetric segmentation architectures—U-Net, Residual U-Net, Attention U-Net, UNETR, and Swin-UNETR—under standardized preprocessing, training, and evaluation protocols. Residual U-Net achieves the highest macro-averaged foreground (Macro-FG) F1 score in all three resolution bins, while helices are segmented more accurately than sheets and NPSS regions. All five architectures achieve their highest Macro-FG F1 scores in the highest-resolution bin, whereas performance varies between the two lower-resolution bins across architectures and secondary-structure classes. Overall, this benchmark enables fine-grained analysis of resolution-dependent segmentation performance, supports reproducible method development, and facilitates controlled comparison of future cryo-EM secondary-structure segmentation approaches.

## Graphical abstract

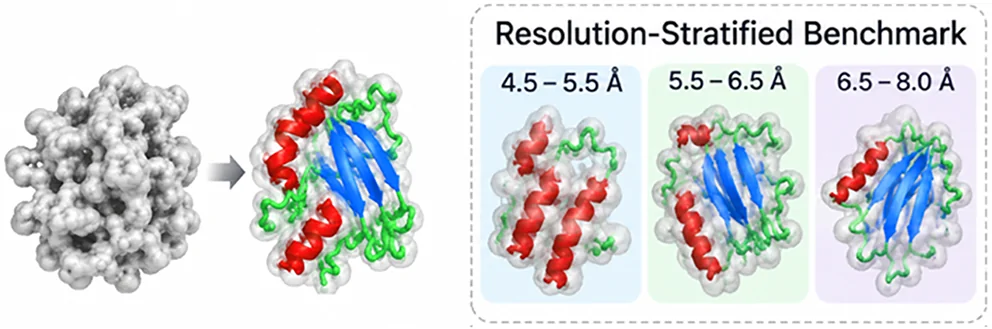

## 1 Introduction

Cryo-electron microscopy (cryo-EM) has emerged as a powerful structural biology technique for visualizing macromolecular complexes across diverse biological systems.^1–3^ Recent advances in cryo-EM reconstruction algorithms and computational processing pipelines have substantially increased the availability of intermediate-resolution density maps suitable for downstream structural analysis.^4–7^ As a result, there is growing interest in developing computational methods that can automatically interpret cryo-EM density maps and facilitate structural characterization of biological macromolecules.

Among biological structure-determination tasks, protein structure modeling is particularly important for understanding protein function and its disruption in disease, as well as for supporting structure-based drug discovery.^8,9^ Accurate segmentation of protein secondary-structure elements, such as helices and sheets, provides important spatial information for protein backbone tracing and atomic-model building from cryo-EM density maps.^10,11^

Despite substantial advances in cryo-EM imaging, automated segmentation of protein secondary structures in intermediate-resolution density maps (approximately 4.5–8 Å, with slightly lower or higher resolution limits used in some studies) remains challenging.^12,13^ Within this resolution range, secondary-structure elements can vary considerably in morphology, continuity, and density characteristics. Although α-helices often appear as elongated, cylindrical, or rod-like density features in intermediate-resolution cryo-EM maps,^14–16^ their density can be locally nonuniform or discontinuous.^17^ β-sheets may exhibit weak strand separation and limited structural clarity, while loop and coil regions often lack distinctive geometric features and well-defined density boundaries. These features generally become less distinguishable at lower resolutions, further complicating accurate secondary-structure segmentation.

Over the years, a range of computational methods has been developed to identify and segment secondary-structure elements directly from high- and intermediate-resolution cryo-EM density maps.^15,18–24^ More recently, deep-learning-based approaches have shown considerable promise for secondary-structure segmentation and related structural interpretation tasks. For intermediate-resolution cryo-EM maps, methods including Emap2sec,^6^ CryoSSESeg,^25^ Emap2sec+,^26^ EMNUSS,^27^ and DeepSSETracer^28^ have demonstrated the potential of deep neural networks to identify and segment protein secondary-structure elements directly from density data. However, these studies were primarily designed to introduce and evaluate specific deep-learning frameworks using study-specific datasets and experimental protocols. Consequently, differences in reported performance cannot be reliably attributed to model architecture because studies also differ in dataset composition, preprocessing, annotation, training, evaluation, and map resolution.

These limitations leave two key questions unanswered: how segmentation performance varies across different ranges within the intermediate-resolution regime and how different architectural paradigms compare under standardized experimental conditions. For example, the relative performance of transformer- and attention-based architectures on secondary-structure segmentation remains largely underexplored in this context. A controlled evaluation framework is therefore needed to compare these architectural paradigms and characterize resolution-associated variations in segmentation performance.

To better understand the effects of cryo-EM map resolution and model architecture on protein secondary-structure segmentation performance, this study introduces a nonredundant, resolution-stratified benchmark dataset together with a controlled evaluation framework for intermediate-resolution cryo-EM maps spanning 4.5–8.0 Å. The dataset provides curated voxel-level annotations, standardized preprocessing, target-chain masking, and predefined partitions for training,validation, and testing. The controlled evaluation framework uses these standardized data conditions to compare representative CNN-based and transformer-based volumetric segmentation architectures. Rather than treating the intermediate-resolution regime as a single resolution range, the dataset is stratified into three resolution bins, enabling systematic assessment of how segmentation performance varies across 4.5–5.5 Å, 5.5–6.5 Å, and 6.5–8.0 Å maps. Evaluating multiple architectures across these resolution ranges within a common evaluation framework provides a controlled basis for comparing their performance.

The primary contributions of this work are summarized as follows:

- We present a nonredundant, resolution-stratified benchmark dataset comprising 972 protein-chain samples from 495 intermediate-resolution cryo-EM structures. The dataset includes DSSP-based voxel-level annotations, target-chain masking, standardized preprocessing, and fixed train/validation/test splits.
- We establish a controlled evaluation framework for comparing representative conventional convolutional, residual, attention-based, and transformer-based volumetric segmentation architectures using common dataset partitions, preprocessing and annotation procedures, and evaluation metrics, together with documented model-training configurations.
- We provide a systematic resolution-wise and class-wise analysis of five representative architectures, characterizing how segmentation performance varies across three intermediate-resolution ranges and among the α-helix, β-sheet, and NPSS classes.

## 2 Related Work and Research Gaps

### 2.1 Detection of Protein Secondary Structures in Intermediate-Resolution Cryo-EM Maps

Helices and sheets provide important structural information for protein backbone tracing and atomic model building from cryo-EM density maps. Therefore, numerous computational methods have been developed to identify and segment these secondary-structure elements directly from cryo-EM density maps. Early methods primarily relied on handcrafted geometric descriptors, density-pattern analysis, template matching, and rule-based feature extraction to detect α-helices and β-sheets in intermediate-resolution density maps.^22,29^

More recently, deep learning has been applied to automated secondary-structure identification. Emap2sec^6^ introduced a three-dimensional convolutional neural network that assigns secondary-structure classes to local regions of intermediate-resolution cryo-EM maps. Emap2sec+^26^ subsequently extended this approach through a residual-network architecture and support for additional macromolecular components. EMNUSS^27^ employed a nested U-Net architecture to annotate α-helices, β-sheets, and other protein regions in both medium- and high-resolution cryo-EM maps. Methods such as CryoSSESeg,^25^ DeepSSETracer,^28^ and DeepSSETracer 2.0 ^30^ have further explored deep-learning-based voxel-level segmentation of protein secondary structures from intermediate-resolution chain-component maps.

Reported quantitative results demonstrate the potential of these methods but also show that secondary-structure identification from experimental intermediate-resolution maps remains challenging. On a common test set of 43 experimental maps spanning 5.0–9.5 Å, the EMNUSS study reported an overall voxel-level F1 score of 0.596 for EMNUSS, compared with 0.480 for Emap2sec and 0.501 for Haruspex when the author-recommended density thresholds were applied.^27^ CryoSSESeg evaluated both voxel- and residue-level segmentation performance on 33 experimental test chains and reported average residue-level F1 scores of 0.76 for helices and 0.60 for β-sheets; its average voxel-level helix F1 scores were 0.71, 0.63, and 0.52 across the three cylindrical fitting-based data-quality bins, respectively.^25^ On a separate dataset of 28 experimental chain-component maps, the DeepSSETracer study reported residue-level F1 scores of 0.72 for helices and 0.65 for β-sheets, compared with 0.77 and 0.42, respectively, for Emap2sec+.^28^ These results indicate that secondary-structure identification remains difficult and that performance varies across structural classes. In particular, the relative performance of DeepSSETracer and Emap2sec+ differed between helix and β-sheet identification. Thus, reliable identification of secondary structures, particularly β-sheets, remains challenging in experimental intermediate-resolution cryo-EM maps.

### 2.2 Research Gaps in Benchmarking and Evaluation

Although a number of studies have explored protein secondary-structure segmentation in intermediate-resolution cryo-EM maps, as discussed above, comparative evaluation of these methods remains challenging because standardized benchmark datasets and evaluation protocols are limited. While some of these methods have been evaluated on common test sets, existing studies differ in dataset composition and size, resolution range, unit of analysis, structural-class definitions, prediction granularity, preprocessing and density-thresholding procedures, annotation strategies, train–test partitioning schemes, and evaluation metrics. For example, some methods process entire maps, whereas others operate on individual chain-component maps; similarly, performance may be evaluated at either the voxel or residue level. Consequently, results reported across studies are not directly comparable, and observed performance differences may reflect variations in experimental design rather than differences among the evaluated methods themselves.

Existing deep-learning studies have primarily evaluated specific architectures using study-specific datasets and experimental protocols. As a result, it remains difficult to determine whether observed performance differences are attributable to model architecture or to differences in data, preprocessing, annotation, training, and evaluation procedures. In addition, systematic evaluation of segmentation performance across finer resolution intervals within the intermediate-resolution regime has not been reported in these studies, leaving the effect of resolution on segmentation performance insufficiently characterized under controlled experimental conditions.

Therefore, addressing these gaps requires not only a curated dataset but also a controlled evaluation framework that combines nonredundant dataset construction with fixed data partitions, standardized voxel-level annotations, consistent preprocessing and training procedures, and common evaluation metrics. Such a framework is necessary to separate architecture-related effects from those arising from experimental design and to systematically quantify resolution- and class-dependent segmentation performance under a unified evaluation setting.

## 3 Materials and Methods

Figure 1 provides an overview of the benchmark dataset construction and partitioning workflow. The following subsections describe each stage of the pipeline in detail.

**Figure 1:**
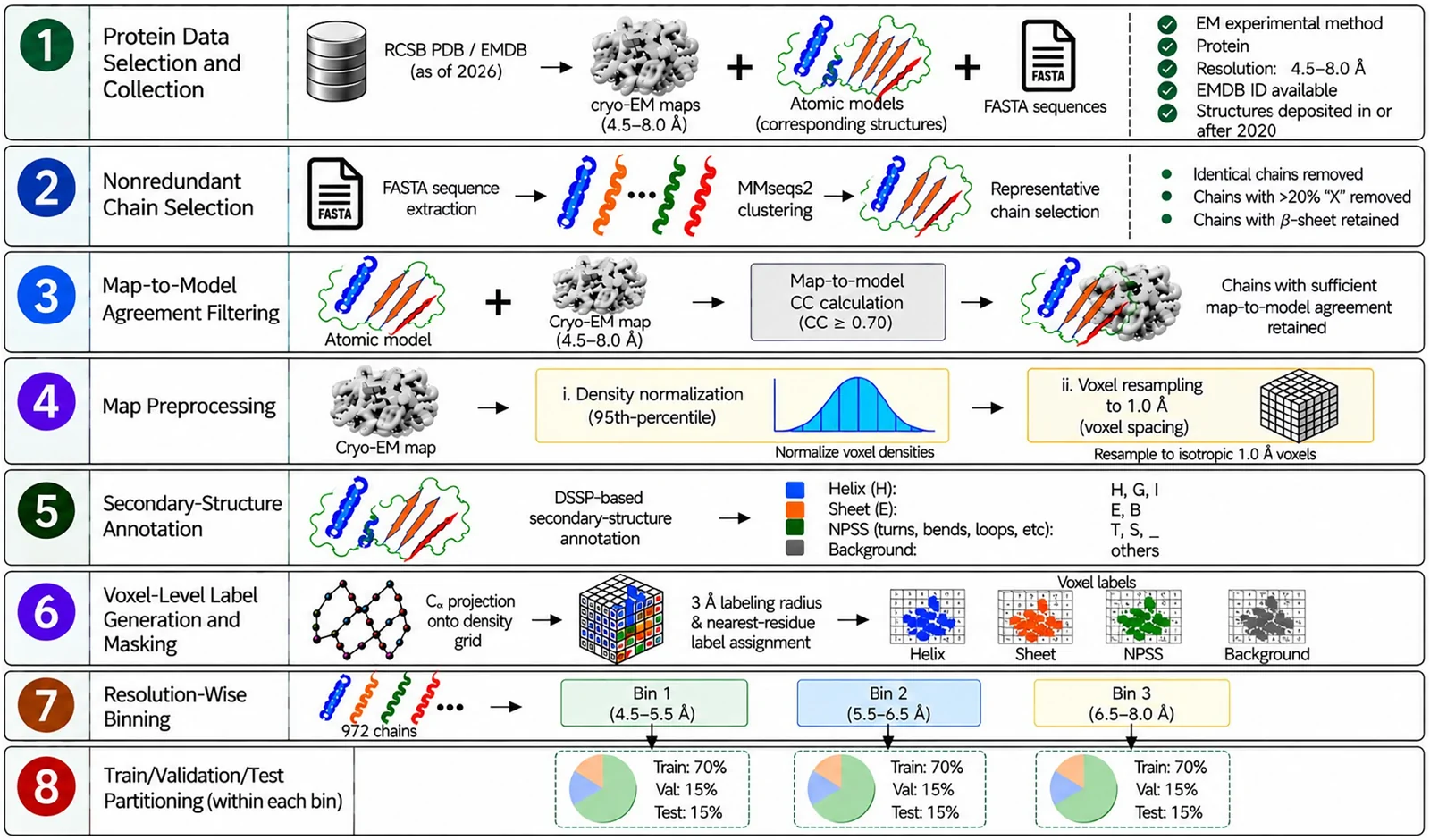
Overview of the benchmark dataset construction, annotation, and partitioning workflow. The workflow includes intermediate-resolution cryo-EM map selection, nonredundant chain selection, map-to-model agreement filtering, map preprocessing, secondary-structure annotation, voxel-level label generation and masking, and resolution-wise train/validation/test partitioning.

### 3.1 Dataset Construction and Cryo-EM Map Preprocessing

#### 3.1.1 Protein Data Selection and Collection

The protein structures investigated in this study were obtained from the RCSB Protein Data Bank (RCSB PDB).^31^ The dataset was constructed using the following selection criteria: (i) Electron Microscopy as the experimental method, (ii) protein as the polymer type, (iii) refinement resolution between 4.5 Å and 8.0 Å, (iv) the presence of an associated EMDB ID, and (v) a PDB release date between January 1, 2020, and January 16, 2026. This release-date range was chosen to construct a contemporary benchmark that reflects recent cryo-EM reconstruction, validation, and model-refinement practices. Limiting the dataset to recent entries reduces variability introduced by older processing workflows while retaining a sufficiently large set of intermediate-resolution structures for resolution-specific evaluation. The database search was performed on January 16, 2026, providing a reproducible snapshot of the available data. Applying these criteria yielded 1099 PDB entries. For each entry, the corresponding PDB atomic model and FASTA file were obtained from theRCSB PDB, while the corresponding cryo-EM density map was obtained from the Electron Microscopy Data Bank (EMDB).^32^

#### 3.1.2 Nonredundant Protein-Chain Selection

Each selected PDB entry was associated with one FASTA file. Therefore, the 1099 selected PDB entries corresponded to 1099 FASTA files, which together contained 4699 distinct protein sequences. When multiple chains in the same PDB entry have identical amino-acid sequences, the FASTA file contains the sequence only once and lists all corresponding chain identifiers in the header of that record. For example, if chains A and B have the same sequence, a single FASTA record represents both chains. Therefore, the number of protein chains represented in the PDB entries exceeds the number of FASTA sequence records. To reduce redundancy and prevent potential data leakage, one chain was initially selected from each identical-sequence group. Afterward, low-quality chains containing more than 20% unknown residues (“X”) were removed. To ensure sufficient representation of β-sheets, chains without β-sheet annotations were excluded. This selection criterion increases the representation of β-sheet-containing chains in the dataset. Accordingly, the benchmark is designed for controlled, class-specific evaluation of helix, sheet, and NPSS segmentation rather than for estimating the prevalence of secondary-structure classes across the broader protein population. After applying these initial filtering criteria, 2097 chains were retained.

To identify sequence redundancy among these 2097 chains, we applied MMseqs2-based clustering.^33^ The chains were compared by sequence similarity and grouped into sequence clusters. A cluster contained chains that met the specified similarity criterion of at least 90% sequence identity over 80% or more of the aligned region. A singleton cluster contained one chain because no other retained chain met this criterion, whereas a non-singleton cluster contained two or more similar chains. This clustering was used to identify groups of redundant or highly similar chains before selecting one representative chain from each group.

The procedure produced 1225 sequence clusters, including 826 singleton clusters and 399 non-singleton clusters. One representative chain was selected from each non-singleton sequence cluster using a hierarchical criterion. The chain with the largest number of annotated β-strands was selected first. If multiple chains had the same number of β-strands, the chain with the larger total number of residues assigned to β-strands was selected. This process, together with the 826 singleton-cluster chains, produced a final nonredundant dataset of 1225 representative protein chains.

#### 3.1.3 Map-to-Model Agreement-Based Filtering

To support reliable voxel-level reference-label generation, we assessed the agreement between each selected protein-chain model and its corresponding cryo-EM density map using cross-correlation (CC) scores computed in ChimeraX.^34^ Because the deposited atomic coordinates were used to generate the voxel-level labels, insufficient model-to-map agreement could result in labels being assigned to spatial locations that are not well supported by the observed density. Prior studies have used model-to-map CC thresholds of approximately 0.65 to identify reliably fitted regions.^12,35^ We therefore applied CC ≥ 0.70 as a conservative quality-control criterion to reduce the likelihood of model-derived annotation errors and obtain higher-confidence reference labels. Of the 1225 selected chains, 1164 satisfied this criterion. The retained chains had CC scores ranging from 0.70 to 0.98, with a mean of 0.87 and a variance of 0.0028 (Figure 2).

**Figure 2:**
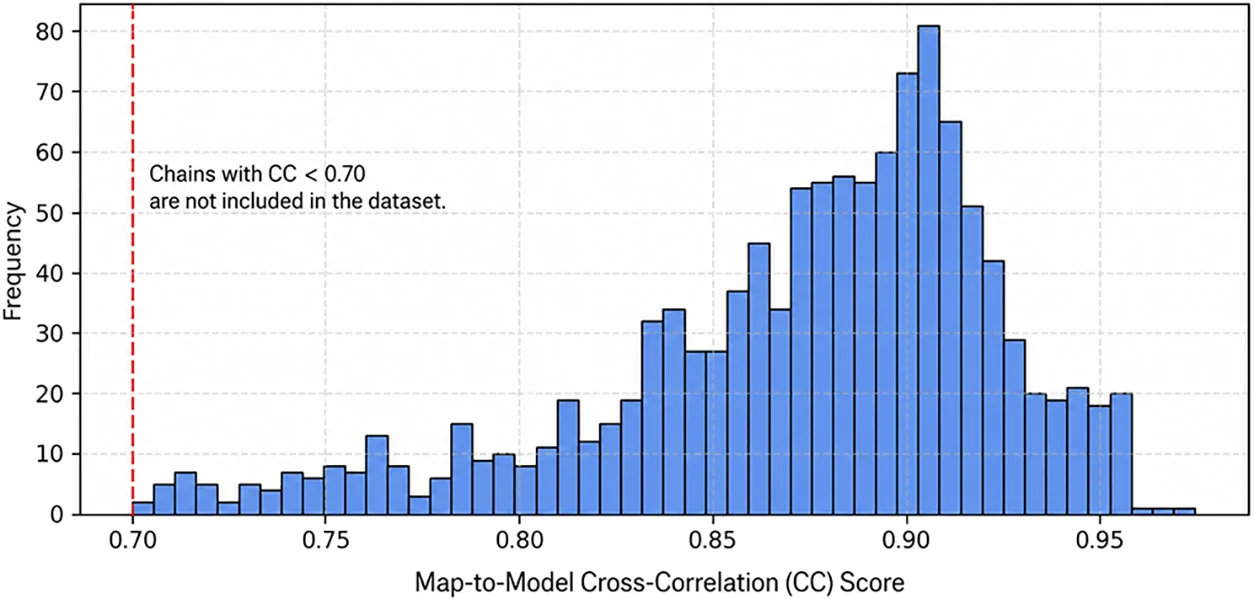
Distribution of cross-correlation (CC) scores for the protein chains that passed the filtering criterion (CC ≥ 0.70).

#### 3.1.4 Map Normalization

To standardize density intensity distributions across cryo-EM maps, percentile-based normalization was applied. Following prior cryo-EM preprocessing practice,^36^ the 95th percentile intensity value was computed for each map and used to scale voxel intensities. This normalization reduces the influence of extreme density outliers while preserving the relative intensity patterns needed for secondary-structure segmentation.

#### 3.1.5 Voxel Resampling

All cryo-EM maps were resampled to a uniform voxel spacing of 1.0 Å using ChimeraX. This step ensured consistent spatial sampling across maps and allowed the segmentation models to process all volumes in a standardized coordinate space. A 1.0 Å voxel spacing has also been used in prior cryo-EM secondary-structure prediction studies.^6,28,37^

### 3.2 Secondary Structure Annotation

For each chain-level cryo-EM density map, voxel-level classes were assigned by combining DSSP-derived secondary-structure annotations with residue coordinate information from the corresponding PDB atomic model. DSSP was used to obtain residue-level secondary-structure annotations from the corresponding PDB model. For each residue, the DSSP annotation was matched to the corresponding residue in the PDB model using the chain identifier and residue identifier. The Cα coordinate and amino acid type were obtained from the PDB model, while the secondary-structure code was obtained from the DSSP output. The combined residue-level information was then mapped onto the voxel grid of the corresponding MRC density map.

The DSSP-derived annotations were grouped into three secondary-structure classes—helix, sheet, and non-periodic secondary structure (NPSS)—with background defined as a fourth voxel-level class. The helix class was defined using DSSP codes H, G, and I, corresponding to α helices, 3_10_ helices, and π helices, respectively. The sheet class was defined using DSSP codes E and B, corresponding to β strands and β bridges. The NPSS class was defined by grouping DSSP codes T and S with residues assigned a blank DSSP secondary-structure code. This category includes turns, bends, loops, and other irregular or non-periodic secondary-structure regions that are not classified as helices or sheets.

To generate voxel-level labels, the physical coordinates of the voxels in each cryo-EM density map were determined from the map origin and voxel spacing. Each voxel was then assigned a secondary-structure label based on its spatial distance to the Cα coordinates of residues in the corresponding atomic model. Voxels within a 3 Å radius of a Cα atom were assigned the secondary-structure class of the corresponding residue. When a voxel was within 3 Å of multiple Cα atoms, the class associated with the nearest Cα atom was assigned, ensuring a unique label for each voxel. Voxels that were not within 3 Å of any Cα atom were assigned to the background class. Although 1164 chains passed the cross-correlation filtering step, DSSP-based annotation failed or produced incomplete chain annotations for 192 chains, which were therefore excluded. The final dataset contained 972 protein-chain samples.

### 3.3 Chain-Specific Cube Extraction and Masking

After voxel-level labeling, each protein chain was processed independently to generate a chain-specific 3D cube. The minimum and maximum voxel indices occupied by the target chain along each spatial axis were used to define its bounding box. The bounding box was padded by 6 voxels in all directions to preserve local structural context and minimize truncation of secondary-structure features near the cube boundaries. Because the cube was extracted using the target chain’s bounding box, secondary-structure regions belonging to neighboring chains could also be present, particularly when the chains were in close spatial proximity.

Each voxel in the cube was associated with a cryo-EM density value, a secondary-structure label, and a binary flag value. Target-chain secondary-structure regions and background voxels were assigned a flag value of 1, whereas secondary-structure regions belonging to neighboring chains were assigned a flag value of 0. During training, the loss was computed over all four classes—background, helix, sheet, and NPSS—for voxels with a flag value of 1, while voxels with a flag value of 0 were excluded from the loss computation. During evaluation, flag values were used in the same manner to exclude neighboring–chain secondary-structure regions from metric computation.

### 3.4 Resolution-Wise Binning and Dataset Partitioning

To evaluate resolution-dependent segmentation performance, the dataset was first divided into three nonoverlapping resolution bins: 4.5 ≤ *r* < 5.5 Å, 5.5 ≤ *r* < 6.5 Å, and 6.5 ≤ *r* ≤ 8.0 Å, where *r* denotes the reported map resolution. For each chain, the voxel counts of helix, sheet, and NPSS regions were computed from the generated labels and used to estimate the relative secondary-structure composition of the chain. Within each resolution bin, a stratified splitting strategy was applied based on this composition to maintain similar label distributions across the training, validation, and test subsets. Protein chains were assigned to the three subsets using an approximate ratio of 70%, 15%, and 15%, respectively. Models were trained and evaluated independently within each resolution bin using the corresponding training, validation, and test splits.

Table 1 summarizes the resolution-wise dataset composition, voxel-level class distribution, and chain-level training, validation, and testing splits. The reported class percentages are voxel-level proportions within the full extracted cubes, which include padded background regions containing no atoms within the 3 Å labeling radius; they therefore differ from residue-level secondary-structure composition. The final dataset contained 972 chain samples from 495 cryo-EM protein structures. Bin 1 contained the largest number of samples, with 539 chains from 259 proteins and a mean resolution of 4.8 Å. Bin 2 contained 206 chains from 112 proteins with a mean resolution of 5.9 Å, while Bin 3 contained 227 chains from 124 proteins with a mean resolution of 7.2 Å.

**Table 1:** Resolution-Wise Summary of Protein Chains, Proteins, Secondary-Structure Voxel Percentages, and Training,Validation, and Testing Splits

Bin	Res. (Å)	Chains	Proteins	Helix voxels%	Sheet voxels%	NPSS voxels%	Train	Val.	Test
Bin 1	4.5–5.5	539	259	6.9	3.9	9.3	379	80	80
Bin 2	5.5–6.5	206	112	7.4	4.1	9.1	147	30	29
Bin 3	6.5–8.0	227	124	6.9	3.7	8.0	161	33	33
Total		972	495				687	143	142

Across the three resolution bins, helix voxels occupied a larger proportion of the labeled regions than sheet voxels. NPSS regions showed the highest voxel percentage among the foreground classes, reflecting the presence of loop, turn, bend, and other non-periodic secondary structure regions in the chain annotations. The chain-level split produced 687 training samples, 143 validation samples, and 142 test samples in total. Specifically, Bin 1 included 379 training, 80 validation, and 80 test chains; Bin 2 included 147 training, 30 validation, and 29 test chains; and Bin 3 included 161 training, 33 validation, and 33 test chains.

### 3.5 Model Architectures and Training Configuration

#### 3.5.1 Deep Learning Architectures

##### 3.5.1.1 3D U-Net

3D U-Net^38^ is a fully convolutional encoder–decoder architecture for volumetric segmentation. The encoder learns hierarchical features through convolution and down-sampling, while the decoder restores spatial resolution through upsampling. Skip connections transfer features between corresponding encoder and decoder stages, combining contextual information with spatial detail.

##### 3.5.1.2 Residual U-Net

Residual U-Net^39^ extends U-Net by incorporating residual blocks into the encoder–decoder architecture. Within each residual block, a shortcut connection combines the block input with the output of the convolutional layers, facilitating information flow and network optimization. Skip connections between corresponding encoder and decoder stages integrate multiscale features for voxel-level segmentation.

##### 3.5.1.3 Attention U-Net

Attention U-Net^40^ extends U-Net by incorporating attention gates into its skip connections. Each gate combines an encoder feature map with a decoder gating signal to generate attention coefficients that modulate the encoder features before they are transferred to the corresponding decoder stage.

##### 3.5.1.4 UNETR

UNETR^41^ combines a Vision Transformer encoder with a convolutional decoder for three-dimensional segmentation. The encoder partitions the input volume into nonoverlapping volumetric patches and processes their position-aware embeddings through transformer blocks. Representations from selected encoder depths are reshaped into multiscale volumetric feature maps and transferred to the decoder through skip connections. The decoder progressively upsamples these features to generate voxel-level predictions.

##### 3.5.1.5 Swin-UNETR

Swin-UNETR^42^ combines a hierarchical Swin Transformer encoder with a U-Net-like convolutional decoder for volumetric segmentation. The encoder uses shifted-window self-attention and patch-merging operations to learn multiscale spatial representations. The decoder progressively upsamples these representations and integrates encoder features through skip connections to generate voxel-level predictions.

#### 3.5.2 Shared Training Configuration

The shared training configuration is summarized in Table 2. All five architectures were trained using the Adam optimizer with an initial learning rate of 3 × 10^–4^. The loss function (*L*) was defined as an equally weighted combination of class-weighted cross-entropy (CE) and Dice loss

L=0.5×weighted
CE+0.5×Dice

**Table 2:** Shared Training Configuration for the Five Evaluated DL Architectures^a^

Parameter	Setting
Input patch size	64^3^
Patch overlap	32^3^
Batch size	16; 4 for Swin-UNETR
Optimizer	Adam
Learning rate	3 × 10^–4^
Loss function	0.5 × Weighted CE + 0.5 × Dice
LR scheduler	ReduceLROnPlateau
Scheduler factor	0.5
Scheduler patience	25
Minimum learning rate	10^–5^
Maximum epochs	250
Early-stopping patience	35

a Batch size was 16 for U-Net, Residual U-Net, Attention U-Net, and UNETR and 4 for Swin-UNETR because of its higher GPU-memory requirement. Additional architecture-specific implementation and parameter details are provided in the Supporting Information (Table S1).

The cross-entropy weights for the background, helix, sheet, and NPSS classes were 0.125, 1.0, 2.25, and 0.7, respectively. A batch size of 16 was used for U-Net, Residual U-Net, Attention U-Net, and UNETR. Swin-UNETR was trained with a batch size of 4 because of its higher GPU-memory requirement. All architectures used the same training, validation, and test partitions, data-augmentation procedures, patch-sampling strategy, and evaluation metrics.

For all architectures, the learning rate was adjusted based on validation loss using a ReduceLROnPlateau scheduler with a reduction factor of 0.5, a patience of 25 epochs, and a minimum learning rate of 10^*–*5^. Training was performed for a maximum of 250 epochs. Early stopping was applied with a patience of 35 epochs based on validation loss.

Data augmentation included random flips along the three spatial axes and rotations about the *x*-, *y*-, and *z*-axes. For each training patch, a rotation angle of 0°, 90°, 180°, or 270° was independently sampled for each axis. The sampled rotations were applied sequentially to both the density volume and its corresponding label volume.

Training patches of size 64^3^ voxels were sampled dynamically from each chain-specific volume. Eight patches were sampled from each volume during each epoch. To reduce background dominance, 70% of the patches were preferentially centered on target-chain foreground voxels, whereas the remaining 30% were sampled randomly from the complete volume. For foreground-biased sampling, a voxel belonging to the helix, sheet, or NPSS class was selected as the initial patch center, followed by a random spatial offset to increase sampling diversity. Sampled patches were checked for the presence of target-chain foreground voxels.

### 3.6 Evaluation Metrics

To assess segmentation performance for the test chains, voxel-level F1 scores were computed for all four classes: background, helix, sheet, and NPSS.^43^ Because the study focused on secondary-structure segmentation, only the class-wise F1 scores for the three foreground classes (helix, sheet, and NPSS) and their unweighted mean, termed Macro-FG F1, were reported. By excluding background, Macro-FG F1 reflects performance on the secondary-structure classes rather than on the dominant background class, which accounted for approximately 80% of the voxels across the three resolution bins. Because evaluation was performed at the individual-chain level, voxels representing secondary-structure regions of neighboring chains within the target-chain bounding box were excluded.

In this voxel-level segmentation setting, the F1 score is equivalent to the Dice coefficient.

For class *c*, the F1 score is defined as

$$F_{1c}=\frac{2TP_{c}}{2TP_{c}+FP_{c}+FN_{c}}$$

where TP*_c_*, FP*_c_*, and FN*_c_* denote the true-positive, false-positive, and false-negative voxel counts for class *c*, respectively. A higher F1 score indicates better agreement between the prediction and ground truth.

In addition to the class-wise F1 scores, Macro-FG F1 was computed as the unweighted mean of the F1 scores for the three foreground secondary-structure classes: helix, sheet, and NPSS

$$\mathrm{Macro}‐\mathrm{FG}\;F1=\frac{F1_{\mathrm{helix}}+F1_{\mathrm{sheet}}+F1_{\mathrm{NPSS}}}{3}$$

For each resolution bin, the reported class-wise and Macro-FG F1 scores were calculated by averaging the corresponding per-chain scores across all test chains in that bin.

## 4 Experimental Results

### 4.1 Overall Segmentation Performance

Figure 3 summarizes the segmentation performance of U-Net, Residual U-Net, Attention U-Net, UNETR, and Swin-UNETR across the three resolution bins. Residual U-Net achieved the highest test Macro-FG F1 score in each bin. In Bin 1 (4.5–5.5 Å), Residual U-Net achieved a Macro-FG F1 score of 0.386, followed by U-Net (0.373), Attention U-Net (0.360), Swin-UNETR (0.332), and UNETR (0.278). In Bin 2 (5.5–6.5 Å), Residual U-Net again achieved the highest score of 0.289, followed by U-Net (0.253), Swin-UNETR (0.234), Attention U-Net (0.229), and UNETR (0.219). In Bin 3 (6.5–8.0 Å), Residual U-Net achieved the highest score of 0.256, followed by Attention U-Net (0.241), U-Net (0.239), Swin-UNETR (0.233), and UNETR (0.183).

**Figure 3:**
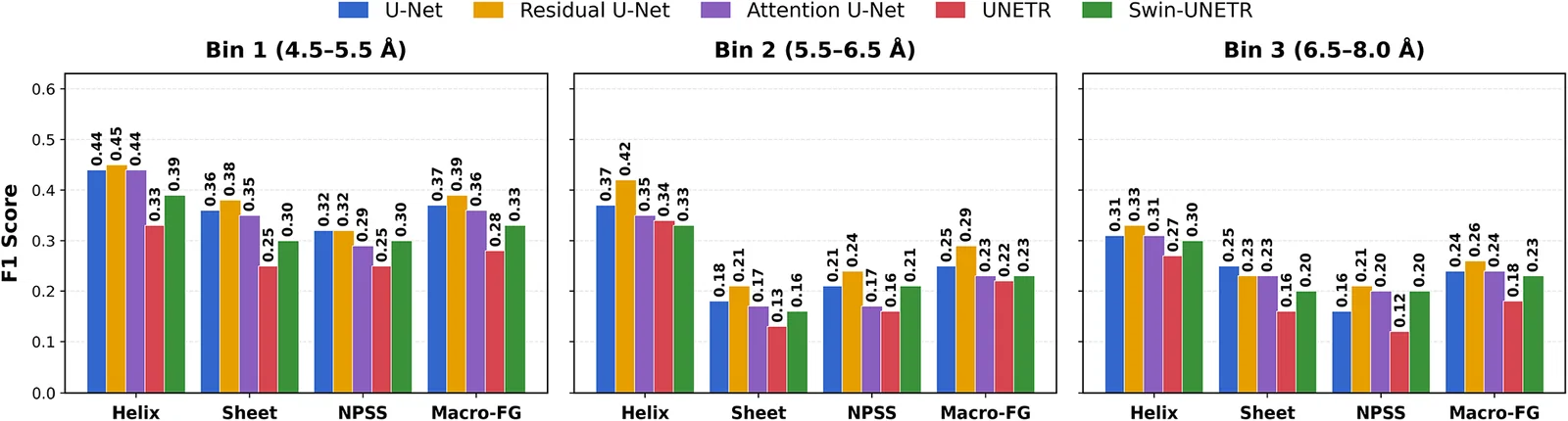
Class-wise segmentation performance across resolution bins. Class-wise test F1 scores and Macro-FG F1 are shown for all five architectures. Across all architectures and resolution bins, helix F1 scores were consistently higher than sheet and NPSS F1 scores.

### 4.2 Class-Wise Segmentation Performance

Figure 3 presents the test F1 scores for helix, sheet, and NPSS across the five architectures and three resolution bins. Across all architecture–bin combinations, helix F1 scores were higher than the corresponding sheet and NPSS F1 scores.

Helix F1 scores ranged from 0.27 to 0.45, with the maximum value occurring for Residual U-Net in Bin 1. From Bin 2 to Bin 3, helix F1 decreased for all five architectures. Sheet F1 scores ranged from 0.13 to 0.38, with the maximum value occurring for Residual U-Net in Bin 1. Sheet F1 increased from Bin 2 to Bin 3 for all five architectures, although the Bin 3 values remained below the corresponding Bin 1 values. NPSS F1 scores ranged from 0.12 to 0.32. From Bin 2 to Bin 3, NPSS F1 decreased for U-Net, Residual U-Net, UNETR, and Swin-UNETR but increased modestly for Attention U-Net. Sheet F1 exceeded NPSS F1 in some architecture–bin combinations, whereas NPSS F1 was higher in others.

Overall, helix F1 scores were consistently higher than sheet and NPSS F1 scores. The most consistent non-monotonic class-specific pattern was the increase in sheet F1 from Bin 2 to Bin 3 across all five architectures.

### 4.3 Performance across Resolution Bins

Figure 4 presents the test Macro-FG F1 scores of the five architectures across the three resolution bins. All five architectures achieved their highest Macro-FG F1 scores in Bin 1. U-Net achieved scores of 0.373, 0.253, and 0.239 in Bins 1, 2, and 3, respectively. Residual U-Net achieved corresponding scores of 0.386, 0.289, and 0.256; Attention U-Net achieved 0.360, 0.229, and 0.241; UNETR achieved 0.278, 0.219, and 0.183; and Swin-UNETR achieved 0.332, 0.234, and 0.233.

**Figure 4:**
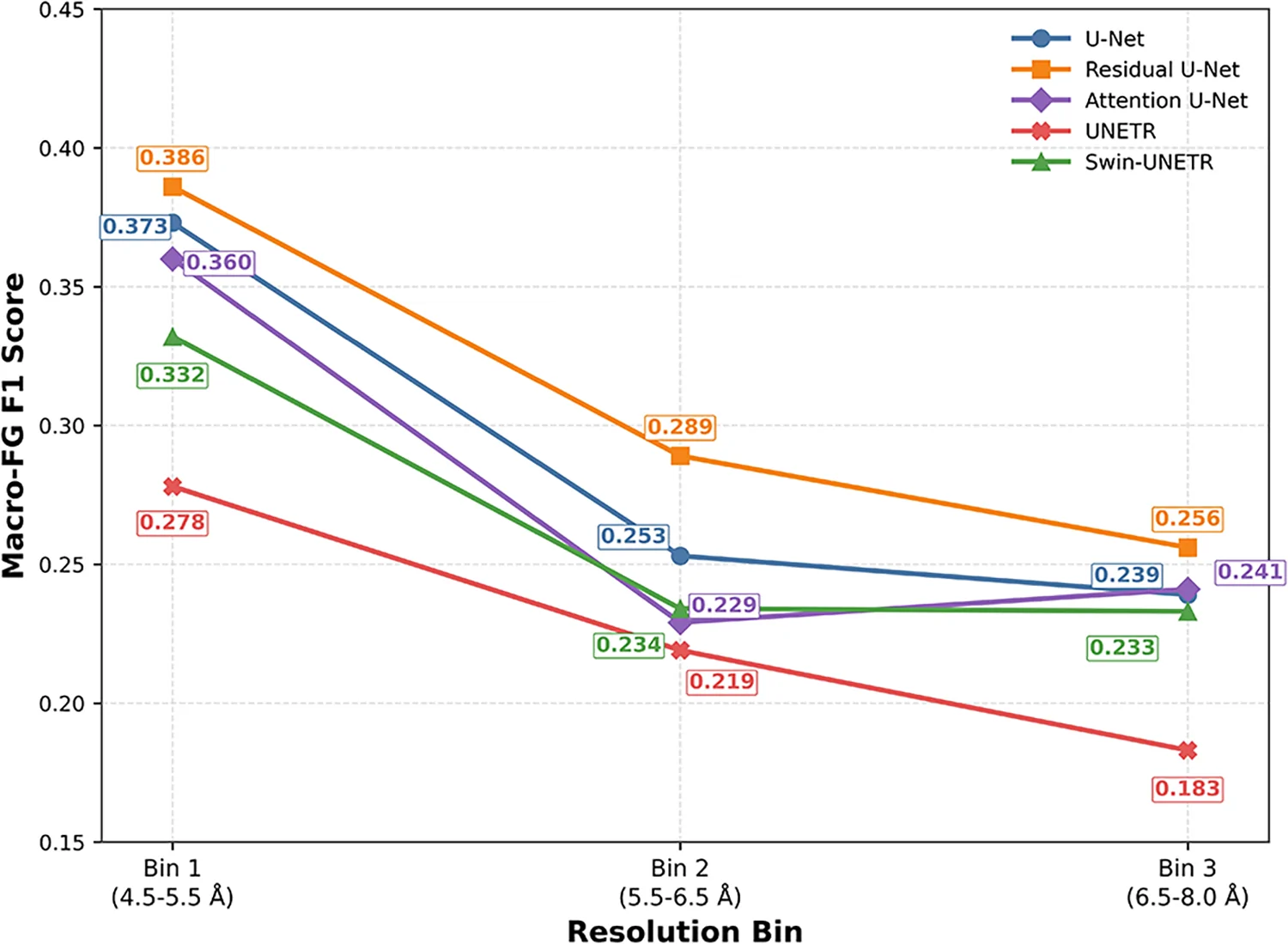
Foreground segmentation performance across different resolution bins. Test Macro-FG F1 scores are shown for all five architectures across the three resolution bins. For all architectures, the highest Macro-FG F1 score was observed in Bin 1, while the relative performance of the architectures varied in Bins 2 and 3.

Macro-FG performance decreased from Bin 1 to Bin 2 for all five architectures. From Bin 2 to Bin 3, Macro-FG F1 decreased for U-Net, Residual U-Net, UNETR, and Swin-UNETR but increased modestly for Attention U-Net. Therefore, overall foreground segmentation performance was consistently highest in Bin 1, whereas changes between Bins 2 and 3 were not monotonic and varied across architectures.

### 4.4 Qualitative Segmentation Examples

For qualitative visualization, one representative test chain was selected from each resolution bin. For each chain, the Macro-FG F1 scores were averaged across the five architectures, and the chains within each bin were ranked according to these model-averaged scores. The median-ranked chain was selected initially. If its predictions were not sufficiently clear for visual comparison, the next higher-ranked chain was selected. This procedure yielded 8U5B chain A, 8IGQ chain B, and 6UVT chain A for Bins 1, 2, and 3, respectively.

Figure 5 presents the segmentation results for these three chains. The left panel for each chain shows the cryo-EM density map with the reference atomic model, while the remaining panels show predictions from the five evaluated architectures. The values below each prediction report the chain-level F1scores for helix, sheet, and NPSS and their unweighted mean, reported as Macro-FG F1.

**Figure 5:**
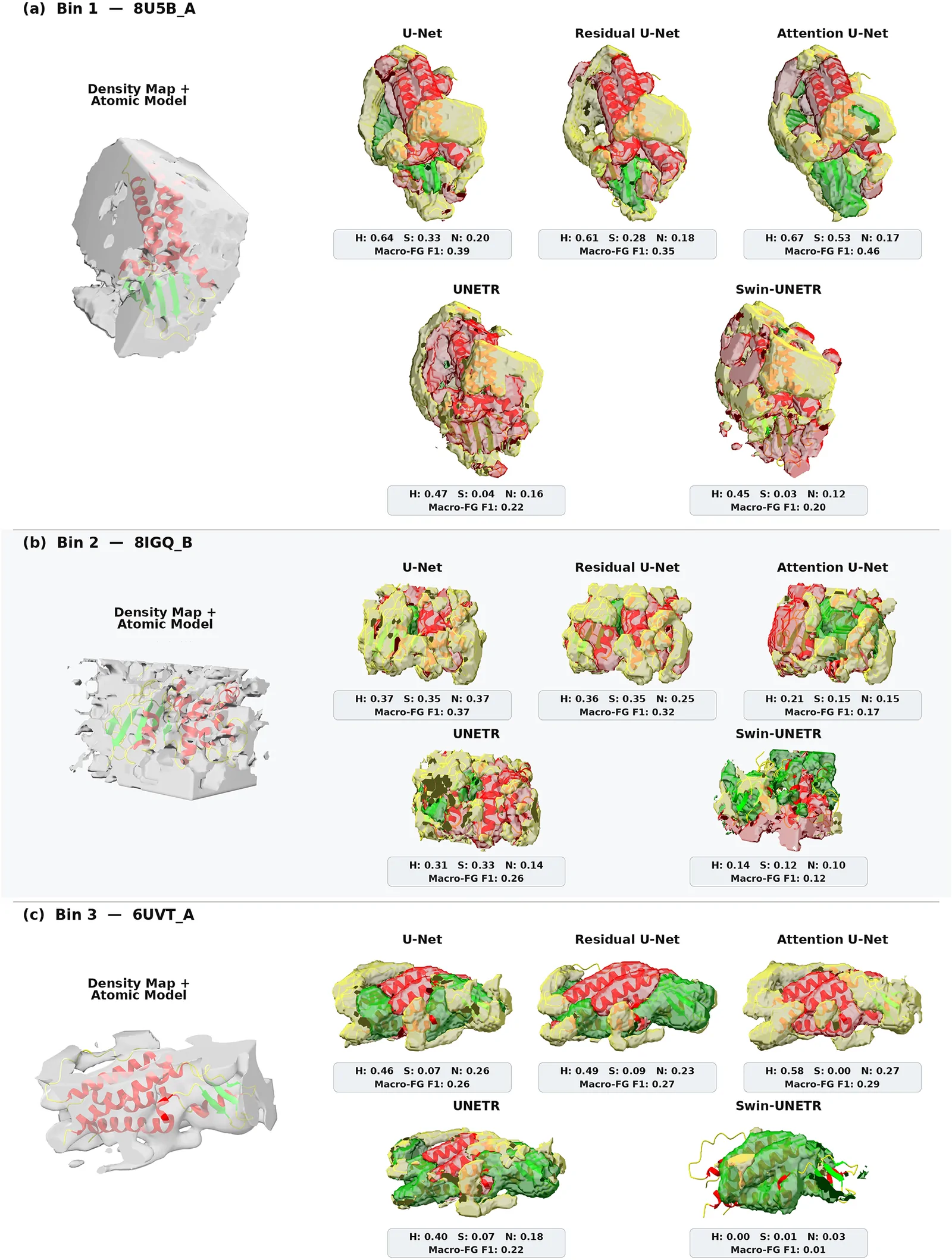
Qualitative predictions for representative chains from (a) Bin 1, 8U5B chain A; (b) Bin 2, 8IGQ chain B; and (c) Bin 3, 6UVT chain A. The left panels show the density maps with reference atomic models, while the remaining panels show semi-transparent predictions from the five architectures. Red, green, and yellow denote helix, sheet, and NPSS, respectively, in both the predictions and reference models. Mismatched classes produce blended colors (e.g., yellow over red appears orange). Values report F1 scores for helix (H), sheet (S), and NPSS (N), along with their unweighted mean (Macro-FG F1).

For the Bin 1 example, Attention U-Net achieved the highest Macro-FG F1 score (0.46), followed by U-Net (0.39), Residual U-Net (0.35), UNETR (0.22), and Swin-UNETR (0.20). Attention U-Net achieved Dice scores of 0.67, 0.53, and 0.17 for helix, sheet, and NPSS, respectively.

For the Bin 2 example, U-Net achieved the highest Macro-FG F1 score (0.37), followed by Residual U-Net (0.32), UNETR (0.26), Attention U-Net (0.17), and Swin-UNETR (0.12). U-Net achieved Dice scores of 0.37, 0.35, and 0.37 for helix, sheet, and NPSS, respectively.

For the Bin 3 example, Attention U-Net achieved the highest Macro-FG F1 score (0.29), followed by Residual U-Net (0.27), U-Net (0.26), UNETR (0.22), and Swin-UNETR (0.01). Attention U-Net achieved a helix Dice score of 0.58 and an NPSS Dice score of 0.27 but a sheet Dice score of 0.00. Swin-UNETR achieved Dice scores close to zero for all three foreground classes in this example.

The architecture with the highest chain-level Macro-FG F1 score therefore varied among the three representative examples. Attention U-Net achieved the highest score for the Bin 1 and Bin 3 examples, whereas U-Net achieved the highest score for the Bin 2 example. Residual U-Net did not achieve the highest score for these individual examples, although it achieved the highest mean Macro-FG F1 across the complete test set in all three resolution bins.

## 5 Discussion

### 5.1 Resolution-Dependent Segmentation Challenges

Across all five architectures, the highest Macro-FG F1 scores were obtained in Bin 1 (4.5–5.5 Å), and Macro-FG F1 decreased from Bin 1 to Bin 2 (5.5–6.5 Å). Changes between Bins 2 and 3, however, varied across architectures. Macro-FG F1 decreased from Bin 2 to Bin 3 for U-Net, Residual U-Net, UNETR, and Swin-UNETR but increased modestly for Attention U-Net. Thus, although foreground segmentation performance was consistently highest in the highest-resolution bin, the results did not show a strictly monotonic decrease across the two lower-resolution bins.

The stronger performance in Bin 1 may be partly explained by the greater visibility of characteristic secondary-structure density patterns at higher resolution. As resolution decreases, the density features associated with α-helices and β-sheets generally become less distinct, reducing the structural information available for voxel-level classification. This loss of structural detail may contribute to the lower Macro-FG F1 scores observed in Bins 2 and 3 relative to Bin 1.

The class-specific results demonstrate that the bin-wise changes were not uniform across secondary-structure classes. From Bin 2 to Bin 3, helix F1 decreased for all five architectures, whereas sheet F1 increased for all five. NPSS F1 decreased for U-Net, Residual U-Net, UNETR, and Swin-UNETR but increased modestly for Attention U-Net. The increase in sheet F1 therefore represents a class-specific pattern rather than a general improvement in segmentation performance in Bin 3. The same annotation, preprocessing, training, and evaluation procedures were applied across all bins, making it unlikely that the observed pattern resulted from a bin-specific procedural difference. However, the resolution bins contain different nonredundant protein chains rather than the same structures observed at multiple resolutions. Differences in structural composition, secondary-structure morphology, and density characteristics among the independently composed bins may therefore contribute to the observed non-monotonic sheet results.

To examine whether the stronger Bin 1 performance was influenced by its larger training set, we performed an additional control experiment using Residual U-Net, the best-performing architecture. The Bin 1 training set was reduced to a stratified subset of 150 chains, while the original validation and test sets were retained. The reduced-training model achieved a Macro-FG F1 score of 0.345, compared with 0.386 when the full Bin 1 training set was used. Although performance decreased with fewer training samples, it remained higher than the corresponding Residual U-Net scores in Bin 2 (0.289) and Bin 3 (0.256). This result indicates that the stronger Bin 1 performance cannot be attributed solely to its larger training set.

Overall, the results show that segmentation performance is influenced by resolution but does not necessarily vary monotonically across independently composed resolution bins. The observed class-specific differences emphasize the importance of evaluating secondary-structure classes separately and interpreting nominal resolution together with the structural characteristics of the samples within each bin.

### 5.2 Class-Specific Segmentation Challenges

The class-specific results showed that helix F1 scores were consistently higher than those for sheet and NPSS F1 scores across all five architectures and resolution bins. However, no consistent ordering was observed between sheet and NPSS F1 scores: sheet F1 was higher in some architecture–bin combinations, whereas NPSS F1 was higher in others. This variation indicates that the segmentation difficulty of these two classes depends on both the architecture and the characteristics of the samples within each resolution bin.

The comparatively higher helix F1 scores may reflect the generally regular and well-defined geometry of helical structures. At intermediate resolution, helices often appear as elongated and continuous density regions that retain recognizable structural patterns. These characteristics provide relatively consistent spatial cues for voxel-level segmentation across different architectures.

In comparison, β-sheet density can exhibit less distinctive structural patterns in intermediate-resolution cryo-EM maps. Individual strands may not be clearly separated, and sheet regions can vary in curvature, extent, and density continuity. These characteristics can make sheet boundaries difficult to distinguish from neighboring NPSS regions and background density, contributing to the lower sheet F1 scores relative to helices. To ensure adequate representation of the sheet class for class-specific evaluation, chains without sheet annotations were excluded during dataset construction. By design, this selection criterion enriched the benchmark with sheet-containing chains. Accordingly, the reported class proportions reflect the benchmark sampling strategy and should not be interpreted as estimates of the prevalence of helix, sheet, and NPSS regions across proteins generally.

NPSS regions present a different segmentation challenge because the class encompasses loops, turns, bends, and other non-periodic structures with substantial variability in geometry and density appearance. Unlike helices, NPSS regions do not exhibit a single recurring structural pattern. Nevertheless, NPSS F1 was not consistently lower than sheet F1, indicating that structural heterogeneity alone does not determine their relative segmentation difficulty under all experimental conditions. Future work could investigate NPSS-focused hard-example sampling, which prioritizes patches containing NPSS voxels that the model repeatedly misclassifies as sheet or background.

Overall, the results demonstrate that secondary-structure classes present distinct segmentation challenges. Helix density provides the most consistent structural cues, whereas the relative difficulty of sheet and NPSS segmentation varies across architectures and resolution bins. These findings support reporting class-specific metrics in addition to an aggregate foreground score.

### 5.3 Benchmark Evaluation of Representative CNN-Based and Transformer-Based Architectures

The five evaluated architectures represent conventional convolutional, residual, attention-gated, and transformer-based approaches to volumetric segmentation. Their evaluation under common dataset partitions, preprocessing procedures, annotations, and metrics demonstrates how the proposed benchmark can support controlled comparison across different architectural paradigms.

Residual U-Net produced the highest Macro-FG F1 score in all three resolution bins. Its higher Macro-FG F1 scores than the standard U-Net across all three bins suggest that residual connections may provide better feature propagation and optimization. However, this comparison does not establish whether the observed performance difference is attributable solely to the use of residual connections.

UNETR ranked last in all three bins, while the relative performance of U-Net, Attention U-Net, and Swin-UNETR varied across resolution bins. U-Net achieved the highest Macro-FG F1 among these three models in Bins 1 and 2, whereas Attention U-Net ranked highest in Bin 3. Attention gating therefore did not consistently improve performance over the standard U-Net, with an improvement observed only in Bin 3 and by a margin of 0.002. These results suggest that the added architectural complexity of attention-based and transformer-based models does not necessarily translate into improved segmentation performance under the dataset sizes and training conditions considered here. The results may reflect the effectiveness of convolutional inductive biases for capturing localized density patterns associated with secondary structures, while transformer-based architectures may require sufficient training data and careful model configuration and optimization to achieve comparable performance.

These results should therefore be interpreted as reference performance for the models considered here rather than as evidence that one architectural family is generally superior. By comparing these architectures under the same data partitions, annotations, training conditions, and evaluation metrics, the study provides a controlled assessment of their relative performance across resolution bins.

### 5.4 Challenges in Cross-Study Comparison

The quantitative results reported by previous secondary-structure identification methods provide important context, but they are not directly comparable with the results obtained using the present benchmark. Existing studies differ in dataset composition and size, resolution range, unit of analysis, structural-class definitions, annotation procedures, preprocessing and density-thresholding methods, prediction granularity, evaluation regions, data partitions, and performance metrics. Although controlled comparisons have been conducted within individual studies, these differences limit comparisons across the literature.

Potential overlap between the training data of previously published methods and the protein chains included as test data in the present benchmark presents an additional complication. Evaluating an existingmethod on chains it may have encountered during its original training could introduce data leakage and lead to an unfair assessment of its performance. A controlled architectural comparison would require each method to be retrained using the same nonredundant training partitions and evaluated using the same target-chain masks, class definitions, and voxel-level metrics. Such retraining is not always possible because implementations, training data, or compatible outputs may not be available.

The present benchmark also differs from earlier evaluation settings by dividing maps spanning 4.5–8.0 Å into three narrower resolution bins and evaluating models independently within each bin. It uses individual protein chains as the unit of analysis and reports Macro-FG F1, which assigns equal weight to helix, sheet, and NPSS F1 scores. Previous studies may instead evaluate complete maps or protein components and report overall or weighted voxel-level F1, residue-level accuracy, or segment-level measures. Accordingly, previously reported values should be interpreted as contextual evidence rather than direct numerical baselines for the present results. By providing fixed nonredundant partitions, standardized annotations, target-chain masking, and common evaluation metrics, the proposed benchmark establishes the conditions needed for controlled comparison of future methods.

### 5.5 Implications for Secondary-Structure Segmentation in Cryo-EM Maps

The findings have several implications for the development and evaluation of secondary-structure segmentation methods. First, the observed performance differences among bins within the intermediate-resolution regime demonstrate the value of evaluating models across finer resolution ranges rather than assuming uniform performance across a broad resolution category. More generally, evaluations conducted within high-, intermediate-, or low-resolution regimes may benefit from finer resolution stratification to identify within-category variation in model performance.

Second, nominal map resolution should not be interpreted independently of the structural and density characteristics of the evaluated samples. Because the resolution bins contained different nonredundant protein chains, differences in secondary-structure composition, morphology, and density appearance may have contributed to the observed performance variation. Evaluations across resolution ranges should therefore consider both nominal resolution and sample composition.

Third, increased architectural complexity or the use of attention and transformer mechanisms did not consistently improve segmentation under the evaluated conditions. Architectural performance depends on the specific implementation, training configuration, and characteristics of the available data. Accordingly, the reported results should be interpreted as reference baselines for the evaluated implementations and experimental conditions rather than as evidence that any architectural family is generally superior.

Fourth, aggregate foreground metrics should be accompanied by class-specific results. A single summary score can obscure differences in performance among helix, sheet, and NPSS regions, particularly when improvement in one class coincides with weaker performance in another. Evaluation should therefore report both aggregate and class-specific performance across resolution ranges.

The proposed benchmark supports examination of these considerations through fixed nonredundant data partitions, standardized voxel-level annotations, target-chain masking, and common evaluation metrics. These features provide a controlled basis for determining whether future methodological improvements generalize across resolution ranges and secondary-structure classes. The benchmark-construction procedure could also be extended to higher- or lower-resolution maps. Although voxel-level label generation may require resolution-specific adjustment and validation, the same controlled training and evaluation framework can be applied once reliable annotations are available.

## 6 Summary and Conclusion

This study presented a nonredundant, resolution-stratified benchmark dataset, established a controlled evaluation framework, and systematically analyzed the resolution-dependent performance of representative deep learning architectures for protein secondary-structure segmentation in intermediate-resolution cryo-EM density maps. The dataset contains 972 protein-chain samples from 495 cryo-EM protein structures resolved at 4.5–8.0 Å and includes voxel-level annotations for helix, sheet, and NPSS regions. The framework combines standardized preprocessing, DSSP-based annotation, target-chain masking, fixed training, validation, and test partitions, documented model-training configurations, and common evaluation metrics to support reproducible assessment of deep learning-based secondary-structure segmentation methods.

Using this framework, we evaluated five representative volumetric segmentation architectures: U-Net, Residual U-Net, Attention U-Net, UNETR, and Swin-UNETR. Residual U-Net produced the highest Macro-FG F1 score in all three resolution bins. All five architectures produced their highest Macro-FG F1 scores in Bin 1, whereas changes between Bins 2 and 3 varied across architectures and secondary-structure classes. Helix F1 scores were consistently higher than than the F1 scores for sheet and NPSS. Sheet F1 exceeded NPSS F1 in some architecture–bin combinations, whereas NPSS F1 was higher in others. These findings indicate that segmentation performance does not vary strictly monotonically with nominal resolution and that aggregate foreground scores alone do not fully characterize class-specific model behavior.

Overall, the proposed benchmark establishes a reusable data resource and reference baselines for conventional convolutional, residual, attention-gated, and transformer-based segmentation architectures. By standardizing dataset construction, annotations, data partitions, preprocessing, and evaluation across resolution ranges and secondary-structure classes, the benchmark provides a common foundation for determining whether future methodological improvements are consistent across experimental conditions. This framework enables more rigorous and reproducible development and evaluation of deep learning methods for secondary-structure segmentation in intermediate-resolution cryo-EM maps.

## Supplementary Material



## Data Availability

The cryo-EM density maps and corresponding atomic models used in this study are publicly available from the Electron Microscopy Data Bank (EMDB) and the RCSB Protein Data Bank (RCSB PDB), respectively. The EMDB and PDB accession identifiers, selected protein chains, benchmark metadata, voxel-level annotations, target-chain masks, and fixed training, validation, and test splits will be made publicly available upon publication at https://github.com/sazzadcsedu/ProteinSecondaryStructureBenchmark.git.

## Notes

Table provides architecture-specific implementation and training details for the five evaluated models (PDF)
