# Supplementary material for: Deep Learning for Protein Secondary-Structure Segmentation in Intermediate-Resolution Cryo-EM Maps: A Resolution-Stratified Benchmark Dataset and Controlled Evaluation
Source: ACS Omega. 2026 Oct 15;11(42):65152–63. doi: 10.1021/acsomega.6c06887 (PMC13717664; doi:10.1021/acsomega.6c06887)
Supplement: Supplementary file 1 [file ao6c06887_si_001.pdf]

Table: Architecture-specific implementation and training details of the evaluated models. A dash indicates that the attribute was not used or was not applicable.

**Attribute**	**U-Net**	**Residual U-Net**	**Attention U-Net**	**UNETR**	**Swin-UNETR**
Architecture	CNN encoder–decoder	Residual CNN encoder–decoder	Attention-gated CNN encoder–decoder	ViT encoder with CNN decoder	Hierarchical Swin encoder with CNN decoder
Encoder stages or blocks	4 stages	4 residual blocks	4 stages	12 transformer blocks	4 hierarchical stages
Encoder feature dimensions	32, 64, 128	16, 32, 64	32, 64, 128	768	Base size 48; stage dimensions 48, 96, 192, 384
Bottleneck dimension	256	128	256	768	768
Convolutional kernel sizes	3^3	3^3	3^3	3^3	3^3
Normalization	Group	Batch	Group	Batch	LayerNorm + InstanceNorm
Residual or attention mechanism	–	Residual shortcut in each block	Attention gate at each skip connection	Multi-head self-attention	Window-based self-attention
Internal patch-embedding size	–	–	–	16^3	2^3
Hidden and MLP dimensions	–	–	–	768 / 2048	48 – 384 / 192–1536
Transformer depth and heads	–	–	–	12 / 12	(2,2,2,2) / (3,6,12,24)
Window size and positional representation	–	–	–	— / Learnable positional embeddings	7×7×7 / Relative position bias
Skip connections	Corresponding encoder stages	Corresponding encoder stages	Attention-gated encoder features	Selected transformer layers: 3, 6, 9, 12	Four encoder stages
Decoder organization	CNN decoder	Transposed convolutions and CNN blocks	CNN decoder	Progressive CNN decoder	U-Net-like CNN decoder
Decoder feature dimensions	128, 64, 32	64, 32, 16	128, 64, 32	512, 256, 128, 64	384, 192, 96, 48
Upsampling method	Transposed convolution	Transposed convolution	Transposed convolution	Transposed convolution	Transposed convolution
Output layer	$1 \times 1 \times 1$ convolution	$1 \times 1 \times 1$ convolution	$1 \times 1 \times 1$ convolution	$1 \times 1 \times 1$ convolution	$1 \times 1 \times 1$ convolution
Model-specific training and regularization					
Gradient-accumulation steps	–	–	–	–	1
Deep-supervision weight	–	–	–	–	0.05
Dropout rate	–	–	–	–	0.1
Attention-dropout rate	–	–	–	–	0.1
Drop-path rate	–	–	–	–	0.2
Trainable parameters	5.60 M	6.25 M	22.67 M	146.25 M	62.19 M
Implementation framework and version	PyTorch 2.10.0 (CUDA 12.8)	PyTorch 2.10.0 (CUDA 12.8)	PyTorch 2.10.0 (CUDA 12.8)	PyTorch 2.10.0 (CUDA 12.8)	PyTorch 2.10.0 (CUDA 12.8)

The supplementary table provides architecture-specific implementation and training details for the five evaluated models.
